# Genetic Evaluation of Early Growth Traits in Yunnan Semi-Fine Wool Sheep

**DOI:** 10.3390/ani15111512

**Published:** 2025-05-22

**Authors:** Yaqian Wang, Hongyuan Yang, Xiaoqi Zhao, Xiaojun Ni, Yuanchong Zhao, Zhengrong You, Qingwei Lu, Sen Tang, Guobo Quan, Xuefeng Fu

**Affiliations:** 1 College of Animal Science, Xinjiang Agricultural University, Urumqi 830052, China; 2 Xinjiang Key Laboratory of Animal Biotechnology, Key Laboratory of Herbivorous Animal Genetics, Breeding and Reproduction, Ministry of Agriculture and Rural Affairs, Institute of Biotechnology, Xinjiang Academy of Animal Sciences, Urumqi 830011, China; 3 Yunnan Animal Science and Veterinary Institute, Kunming 650224, China; 4 Qiaojia Agriculture and Rural Bureau, Qiaojia 654600, China; 5 Zhaotong Agriculture and Rural Bureau, Zhaotong 657000, China

**Keywords:** Yunnan semi-fine wool sheep, genetic parameters, early growth traits

## Abstract

With economic development and improved living standards, the demand for mutton and wool continues to grow, and improving the production performance and genetic potential of sheep breeds has become the key to promoting the high-quality development of the sheep industry. Thus, this study analyzes the influencing factors of the early production traits of Yunnan semi-fine wool sheep, optimizes the genetic evaluation model, and relies on accurate genetic parameter estimation to provide a theoretical basis for formulating a scientific and efficient breeding strategy for this breed. Data were collected from the Laishishan and Xiaohai breeding farms in Qiaojia, Yunnan, covering production records of the core flock from 2018 to 2022. Using the GLM procedure in SAS 9.4 software, this study analyzed the non-genetic influences on early production traits in Yunnan semi-fine wool sheep. Concurrently, Danish Milk Unit 5 (DMU 5) software estimated the variance components across various animal models for each trait. Employing the Akaike Information Criterion (AIC) and likelihood ratio test (LRT), six models were tested, incorporating or excluding maternal inheritance and environmental impacts, to identify the optimal model for deriving the genetic parameters. The results show that the birth year, dam age, sex, flock and litter size significantly affect both the Birth Weight (BWT) and Weaning Weight (WWT) (*p* < 0.01). Additionally, the birth month was found to exert a significant effect on Birth Weight (BWT) (*p* < 0.01), the weaning month has a significant effect on the Weaning Weight (WWT) (*p* < 0.05). No significant effects of farm location were observed on either trait (*p* > 0.05). The most accurate genetic evaluation model determined the heritability of the Birth Weight (BWT) and Weaning Weight (WWT) as 0.3123 and 0.3471. From a production perspective, improving lamb birth, Weaning Weight (WWT), feed composition, and maternal nutrition during gestation is vital for breeding efficiency. This study not only identified the optimal animal models for early growth traits in Yunnan semi-fine wool sheep, offering a precise basis for estimating genetic parameters but also provides theoretical guidance for genetic selection and breed improvement in this population.

## Simple Summary

The Yunnan semi-fine wool sheep is the first semi-fine wool sheep breed developed in China. This study investigates the non-genetic factors influencing early growth traits and estimates their genetic parameters, providing a scientific basis for the selective breeding of this breed and other semi-fine wool sheep populations. The findings indicate that birth year, dam age, sex, flock and litter size significantly affect Birth Weight (BWT) and Birth Weight (BWT) (*p* < 0.01). Additionally, birth month was found to significantly influence Birth Weight (BWT) (*p* < 0.01), while weaning month had a statistically significant effect on Weaning Weight (WWT) (*p* < 0.05). No significant effects of farm location were observed on either trait (*p* > 0.05). The most accurate genetic evaluation model determined the heritability of the Birth Weight (BWT) and Weaning Weight (WWT) as 0.3123 and 0.3471.

## 1. Introduction

Economic growth and rising living standards have led to an increased demand for mutton and wool, fostering the expansion of the sheep industry [1]. Amid this growing demand, developing high-yield and well-adapted breeds is crucial for ensuring the sustainability and productivity of the sheep industry [2]. The Yunnan semi-fine wool sheep, China’s first semi-fine wool breed, is a dual-purpose breed for both meat and wool production, primarily distributed in Zhaotong, Yunnan Province [3]. This breed was developed in the late 1960s by the Yunnan Academy of Animal Science and Veterinary Medicine through successive crossbreeding of long-wool semi-fine sheep (e.g., Romney and Lincoln) with local coarse-wool sheep. This breeding strategy enabled the Yunnan semi-fine wool sheep to adapt to cold mountainous regions and southern grasslands [4,5]. Due to its strong adaptability and favorable production performance, this breed has become an important genetic resource in the region. As of 2023, the original breeding farm in Qiaojia County maintains a core flock of 1425 breeding sheep (including 1143 at Laishishan Breeding Farm and 282 at Xiaohai Breeding Farm) [6]. The Yunnan semi-fine wool sheep breeding farm primarily focuses on conservation, but the selective breeding of the core flock faces numerous challenges, including limited population size. Otherwise, most of the breeding sheep produced by the original farm are sold as commercial sheep, resulting in low coverage of improved breeds and difficulty in realizing the benefits of the new variety [6,7].

Early growth traits, particularly the Birth Weight and Weaning Weight, are critical indicators in selective breeding, as they directly influence lamb survival, growth rate, and overall production efficiency [8,9]. Selecting for these traits can significantly accelerate genetic progress and improve economic returns. However, achieving genetic improvement requires precise genetic parameter estimation, which serves as the foundation for effective breeding programs [10,11].

Accurate genetic parameter estimation is essential for assessing population characteristics and refining breeding strategies [12]. This requires reliable statistical models with individual additive genetic effects, maternal genetic effects, maternal permanent environmental effects, and a variety of non-genetic factors influencing trait expression [13]. Therefore, selecting an optimal animal model tailored to the specific breeding conditions is crucial for ensuring reliable genetic evaluations and driving sustainable genetic improvement.

Currently, there are limited studies on genetic parameter estimates of Yunnan semi-fine wool sheep; this study analyzes early growth traits using data collected from the Laishishan and Xiaohai Breeding Farms between 2018 and 2022. SAS software was employed to assess non-genetic factors, while variance components were estimated using the Danish Milk Unit 5 (DMU 5) software under a single-trait animal model. The likelihood ratio test (LRT) and Akaike Information Criterion (AIC) were used to compare different models and identify the optimal model for estimating genetic parameters of the Birth Weight and Weaning Weight. This study provides a theoretical foundation for selective breeding, offering key insights into the genetic improvement of Yunnan semi-fine wool sheep.

## 2. Materials and Methods

### 2.1. Data Sources and Processing

The Yunnan semi-fine wool sheep are raised under a grazing-based husbandry system. This study focused on newborn lambs of Yunnan semi-fine wool sheep from the Laishishan and Xiaohai Breeding Farms in Qiaojia, Yunnan, and collected lambing records, mating records, and pedigree data from these two farms during 2018–2022. Microsoft Excel software 2019 was used for data cleaning and preprocessing. Missing values in the raw data were identified and excluded, and extreme or abnormal data were removed based on practical production conditions. After these steps, a total of 3252 complete datasets of early growth traits were obtained. The early growth traits included the Birth Weight and Weaning Weight. Descriptive statistical analysis results for each trait are presented in Table 1.

### 2.2. Stratification of Environmental Fixed Effects

The classification of different factor levels is detailed in Table 2. Based on the actual production conditions and data structure of the sheep farm, the effects of seven factors—the lamb birth year, dam age, birth month, birth type, birth sex, weaning month, and weaning age—on the Birth Weight and Weaning Weight of Yunnan semi-fine wool sheep were analyzed.

### 2.3. Statistical Analysis Methods

The general linear model (GLM) procedure in SAS 9.4 software was used for the least squares analysis of the primary traits [14].

The statistical model for the Birth Weight is as follows:

(1) $$Y_{ijklmcrn}=u+a_{i}+b_{j}+d_{k}+t_{l}+h_{m}+f_{c}+O_{r}+e_{ijklmcrn}$$

where $Y_{ijklmcrn}$ is the observed trait value; *u* is the overall mean; $a_{i}$ is the effect of the *i* level of the birth year; $b_{j}$ is the effect of the *j* level of the birth month; $d_{k}$ is the effect of the *k* level of the birth sex; $t_{l}$ is the effect of the *l* level of the birth type; $h_{m}$ is the effect of the *m* level of the maternal age; $f_{c}$ is the effect of the c level of the farm; $O_{r}$ is the effect of the r level of the herd group; $e_{ijklmcrn}$ is the random residual effect.

The statistical model for the Weaning Weight is as follows:

(2) $$Y_{ijklmcrvn}=u+a_{i}+b_{j}+d_{k}+t_{l}+h_{m}+f_{c}+O_{r}+P_{v}+e_{ijklmcrvn}$$

where $Y_{ijklmcrvn}$ is the observed trait value; *u* is the overall mean; $a_{i}$ is the effect of the *i* level of the birth year; $b_{j}$ is the effect of the *j* level of the weaning month; $d_{k}$ is the effect of the *k* level of the birth sex; $t_{l}$ is the effect of the *l* level of the birth type; $h_{m}$ is the effect of the *m* level of the maternal age; $f_{c}$ is the effect of the c level of the farm; $O_{r}$ is the effect of the r level of the herd group; $P_{v}$ is the effect of the v weaning age days; $e_{ijklmcrvn}$ is the random residual effect.

### 2.4. Genetic Parameter Estimation Model

Six distinct animal models were used for each trait by including or excluding the following effects: individual additive genetic effects, maternal additive genetic effects, maternal permanent environmental effects, and the covariance between direct and maternal additive genetic effects [15]. The models were structured as follows:

(3) $$\mathrm{Model}\;1\;\;Y=X_{b}+Z_{1a}+e$$

(4) $$\mathrm{Model}\;2\;\;Y=X_{b}+Z_{1a}+Z_{3mp}+e$$

(5) $$\mathrm{Model}\;3\;\;Y=X_{b}+Z_{1a}+Z_{2mg}+e\;\;\;\mathrm{COV}(a,m)=0$$

(6) $$\mathrm{Model}\;4\;\;Y=X_{b}+Z_{1a}+Z_{2mg}+e\;\;\;\mathrm{COV}(a,m)=A\sigma_{am}$$

(7) $$\mathrm{Model}\;5\;\;Y=X_{b}+Z_{1a}+Z_{2mg}+Z_{3mp}+e\;\;\;\mathrm{COV}(a,m)=0$$

(8) $$\mathrm{Model}\;6\;\;Y=X_{b}+Z_{1a}+Z_{2mg}+Z_{3mp}+e\;\;\;\mathrm{COV}(a,m)=A\sigma_{am}$$

In the model, *Y* is the observation vector of the traits; *b* represents the fixed effects; *a* denotes the individual additive genetic effects; *mg* refers to the maternal additive genetic effects; *mp* indicates the maternal permanent environmental effects; *e* is the residual effect. *X*, $Z_{1},Z_{2}$, and $Z_{3}\;$ are the incidence matrices corresponding to the fixed effects, individual additive genetic effects, maternal additive genetic effects, and maternal permanent environmental effects, respectively. A represents the additive genetic relationship matrix, which accounts for the covariance ($\sigma_{am}$) between the individual additive genetic effects (a) and maternal additive genetic effects (*mg*).

### 2.5. Comparison of Different Models

To accurately estimate the genetic parameters for different traits of the Yunnan semi-fine wool sheep, this study designed six different animal models for fitting and performance evaluation, in order to identify the optimal animal model for each trait.

The Akaike Information Criterion (AIC) was used to evaluate the variance components estimated by the different models. The formula for calculating AIC is as follows [16]:

AIC = 2k − 2LogL(9)

In the formula, L represents the log-likelihood function value, and K denotes the number of parameters to be estimated [17]. The Akaike Information Criterion (AIC) reflects the impact of the number of parameters to be estimated on the estimation accuracy. While increasing the number of parameters in a model can improve the goodness of fit, an excessive number of parameters can lead to overfitting. Therefore, AIC rewards goodness of fit while penalizing the increase in the number of estimated parameters. When selecting the best model from a set of models, the model with the smallest AIC value is preferred [18], as it provides the best estimation of variance components [19].

The likelihood ratio test was used to compare the relative fit of the different models. The test formula is as follows:

(10) $$\mathrm{LR}=-2\log\frac{L1}{L2}\;=[-2\log\;(L_{1})]-[-2\log\;(L_{2})]$$

In the formula, LR represents the likelihood ratio, and $L_{1}$ and $L_{2}$ represent the log-likelihood values of Model 1 and Model 2, respectively, where Model 1 is a sub-model of Model 2. Additionally, LR follows a chi-square distribution ($\chi^{2}$) with degrees of freedom equal to the difference in the number of estimated parameters between Model 2 and Model 1. If the test result is significant, it indicates that the added parameters have a significant impact on the trait; otherwise, the parameters do not have a significant effect [20,21,22].

### 2.6. Genetic Parameter Calculation

The variance components estimated by the Danish Milk Unit 5 (DMU 5) software were substituted into the following formula to calculate the genetic parameters:

Individual heritability:

(11) $$h_{\alpha }^{2}\;=\sigma_{\alpha }^{2}/\sigma_{p}^{2}$$

Maternal heritability:

(12) $$h_{m}^{2}\;=\sigma_{m}^{2}/\sigma_{p}^{2}$$

Maternal permanent environmental heritability, respectively:

(13) $$h_{c}^{2}=\sigma_{c}^{2}/\sigma_{p}^{2}$$

Phenotypic variance:

(14) $$\sigma_{p}^{2}\;=\sigma_{a}^{2}\;+\sigma_{m}^{2}+\sigma_{e}^{2}$$

where $\sigma_{\alpha }^{2},\sigma_{m}^{2},\sigma_{c}^{2},\;\sigma_{e}^{2}\;$ and $\sigma_{p}^{2}$ represent the direct genetic variance, mother genetic variance, mother permanence environmental variance, residual variance, and direct and mother genetic covariance, $\sigma_{am}$ represents the covariance between individual additive genetic effects and maternal additive genetic effects. $h_{\alpha }^{2},h_{m}^{2}$, and $h_{c}^{2}$ represent individual heritability, maternal heritability, and maternal permanent environmental heritability, respectively.

## 3. Results

### 3.1. Least Squares Analysis of Birth Weight (BWT) and Weaning Weight (WWT) in Yunnan Semi-Fine Wool Sheep

#### 3.1.1. Least Squares Analysis of Birth Weight in Yunnan Semi-Fine Wool Sheep

As shown in Table 3, the birth year, sex, lambing date, flock, litter size, and dam age all had highly significant effects on the Birth Weight (*p* < 0.01). However, there were no significant effects of the farm on the Birth Weight of Yunnan semi-fine wool sheep (*p* > 0.05). The fixed effects of Birth Weight for each trait are shown in Table 4.

#### 3.1.2. Least Squares Analysis of Weaning Weight (WWT) in Yunnan Semi-Fine Wool Sheep

The fixed effects of the Weaning Weight traits are presented in Table 5. As shown, the birth year, sex, weaning age, flock, litter size, and dam age all exhibited highly significant effects on the Weaning Weight of Yunnan semi-fine wool sheep (*p* < 0.01). Additionally, the weaning month had a significant effect on the Weaning Weight (*p* < 0.05), while the farm location had no significant impact on the Weaning Weight (*p* > 0.05). The fixed effects of the Birth Weight for each trait are shown in Table 6.

### 3.2. Estimation of Variance Components for Early Growth Traits in Yunnan Semi-Fine Wool Sheep Using Different Animal Models

#### 3.2.1. Estimation of Variance Components for Birth Weight (BWT) Using Different Animal Models

As shown in Table 7, the heritability estimates for Birth Weight varied across models, with the lowest value observed in Model 5 (0.2430) and the highest in Model 6 (0.3828). Maternal genetic effects also exhibited considerable variation, ranging from 0.1205 in Model 3 to 0.2011 in Model 5. Similarly, maternal permanent environmental effects showed notable differences across the models, ranging from 0.0831 to 0.1295.

#### 3.2.2. Estimation of Variance Components for Weaning Weight (WWT) Using Different Animal Models

As presented in Table 8, the heritability estimates for Weaning Weight varied across the models, with the lowest value observed in Model 5 (0.1429) and the highest in Model 4 (0.6635). Maternal genetic effects also exhibited substantial variation, ranging from 0.0967 in Model 3 to 0.2908 in Model 5. Similarly, maternal permanent environmental effects showed notable differences across the models, ranging from 0.0879 to 0.1298.

### 3.3. Comparison of Different Animal Models

#### 3.3.1. Comparison of Different Models Using Akaike Information Criterion (AIC)

The −2LogL and AIC values for different animal models are listed in Table 9. Based on the AIC analysis, Model 4 provided the best genetic parameter estimates for Birth Weight, indicating that individual additive genetic effects, maternal effects, and the interaction between individual additive genetic effects and maternal additive genetic effects have a significant impact on the Birth Weight. Model 2 provided the best genetic parameter estimates for Weaning Weight, suggesting that individual additive genetic effects and maternal permanent environmental effects significantly influence the Weaning Weight.

#### 3.3.2. Comparison of Different Models Using Likelihood Ratio Test (LRT)

The likelihood ratio and chi-square test results for the comparison of the six models are presented in Table 10. The results show that for Birth Weight in early traits of Yunnan semi-fine wool sheep, no significant differences were observed between Model 2 and Model 6, as well as Model 5 and Model 6 (*p* > 0.05). However, significant differences were found between the other models (*p* < 0.05). For Weaning Weight, no significant differences were found between Model 5 and Model 6 (*p* > 0.05), while significant differences were observed between the other models (*p* < 0.05).

## 4. Discussion

### 4.1. Effects of Different Fixed Effects on Early Growth Traits

This study considered seven fixed effects, including the lamb’s birth year, maternal age, birth month, birth type, birth sex, weaning month, and weaning age. For the Birth Weight of Yunnan semi-fine wool sheep, five factors were primarily considered: birth year, maternal age, birth month, birth type, and birth sex. For the Weaning Weight, the above five factors were included along with the weaning month and weaning age. This study found that the average Weaning Weight of Yunnan semi-fine wool sheep showed an increasing trend over time, with longer weaning periods resulting in a higher Weaning Weight. The birth year, sex, lambing date, herd, litter size, and maternal age influenced the Birth Weight of Yunnan semi-fine wool sheep, while the birth year, sex, weaning age, herd, litter size, and maternal age influenced the Weaning Weight. Bradford [23] suggested that factors such as the breed, nutritional level, litter size, maternal age, and season affect lambs through the ewe. Sabri Gul’s study on Kilis goats found that the sex, birth type, herd, and year influenced early growth traits, which aligns with the findings of this study. Similar results were reported by Yang Jie et al. [24] for the Birth Weight of Chinese Merino (Xinjiang type) lambs, Li Xiuli et al. [25] for the Weaning Weight of South African Meat Merino sheep, and Masala et al. [26] for the average daily gain before weaning in Avikalin sheep.

Yunnan semi-fine wool sheep are raised under grazing conditions, and variations in the birth year may affect traits due to differences in feeding management, feed supply, climate changes, and personnel adjustments on the farm. The influence of the birth season on the Birth Weight, Weaning Weight, and average daily gain before weaning may be attributed to differences in environmental conditions across months. Since Yunnan semi-fine wool sheep are primarily pasture-raised, temperature variations across months could also affect forage moisture and nutritional quality. To improve early growth traits in Yunnan semi-fine wool sheep, ensuring adequate feed supply during the ewe’s gestation period is essential to meet the nutritional needs of both the ewe and the fetus. Additionally, supplementary feeding before and after birth may enhance the lambs’ Birth Weight and Weaning Weight.

### 4.2. Comparison of Variance Component Estimation Across Different Animal Models

The coefficient of variation for the Birth Weight of Yunnan semi-fine wool sheep was 14.27%, a result similar to the 17% coefficient of variation for the Birth Weight reported by Tian Haining [27] of Qinghai Plateau semi-fine wool sheep. In the model by Behrem et al. [28], which considered both individual additive genetic effects and maternal additive genetic effects, the heritability estimates for Birth Weight were 0.13 for Sardi sheep and 0.22 for Central Anatolian Merino sheep. Mohammadi et al. [29] included random effects such as individual additive genetic effects, maternal additive genetic effects, and maternal temporary environmental effects in their model, estimating the heritability of the Birth Weight of Shal sheep at 0.13. Li et al. [30] and Mandal et al. [31] estimated the heritability of the Birth Weight of Australian Merino sheep and Muzaffarnagari sheep at 0.15, which aligns with the findings of this study. However, the heritability estimate in this study (0.3123) is higher than those reported by Behrem et al. (0.13 and 0.22) and Mohammadi et al. (0.13), as well as Li et al. and Mandal et al. (0.15).

The heritability of the Weaning Weight of Yunnan semi-fine wool sheep was 0.3471. Hanford et al. [32] estimated the heritability of the Weaning Weight of Columbia sheep at 0.07, with the model including individual additive genetic effects, maternal additive genetic effects, and maternal permanent environmental effects, all of which indicated low heritability. Jalil-saghale et al. [33] estimated the heritability of the Weaning Weight of Baluchi sheep at 0.12, with the model incorporating individual additive genetic effects, maternal additive genetic effects, and maternal permanent environmental effects. Safari et al. [34] estimated the heritability of the Weaning Weight of Australian Merino sheep at 0.29, with the model including individual additive genetic effects, litter effects, maternal permanent environmental effects, and the interaction between individual direct genetic effects and maternal genetic effects, a result similar to that of this study. Di et al. [35] studied Chinese super-fine Merino sheep and estimated the heritability of the Weaning Weight at 0.16, with the model considering individual additive genetic effects and maternal permanent environmental effects.

In mammals, early growth traits are influenced by both direct additive genetic effects and maternal effects due to the prolonged maternal dependence of offspring [36,37]. Maternal effects, including maternal genetic contributions and maternal environmental influences, can significantly impact lamb growth and survival. Studies on Iranian Baluchi and Munjal sheep emphasize the necessity of incorporating maternal effects into statistical models to enhance the accuracy of genetic parameter estimation [36,38]. If maternal effects are ignored, models may overestimate additive genetic variance, leading to biased heritability estimates and reduced selection efficiency [39]. Accurately partitioning maternal genetic and maternal permanent environmental effects ensures unbiased variance estimation, thereby optimizing breeding strategies and enhancing genetic gain [40,41]. Lambs receive supplemental feeding after birth, and their Weaning Weight may be influenced by feeding management practices, resulting in lower heritability. Variations in growth performance and genetic structure among different sheep breeds can also lead to differences in Weaning Weight, thereby increasing the heritability estimates. Additionally, factors such as feeding management and environmental conditions, including different nutritional levels and growth environments, may contribute to varying degrees of phenotypic variation.

## 5. Conclusions

This study identified optimal animal models for estimating the genetic parameters of early growth traits in Yunnan semi-fine wool sheep, with Model 4 best suited for the Birth Weight and Model 2 for the Weaning Weight. The heritability estimates for these traits were moderate (0.3123) for Birth Weight; (0.3471) for Weaning Weight, indicating substantial genetic potential for selection. The selection for higher birth and Weaning Weight, combined with the strategic culling of older or low-performing ewes based on reproductive performance and maternal traits, can accelerate genetic gain. Implementing these strategies in breeding programs is expected to improve flock productivity, reduce feeding costs, and promote sustainable sheep production.

## Figures and Tables

**Table 1:** Descriptive statistics of valid records for early growth traits and reproductive traits.

Traits	Sample Size	Mean ± SD	Min (KG)	Max (KG)	Coefficient of Variation, %
BWT (kg)	3252	4.39 ± 0.62	2.10	7	14.27
WWT (kg)	3252	21.67 ± 4.28	6.50	46	19.78

**Table 2:** Level division of non-genetic factors for each trait.

Factor								Level
Birth Year	Farm	Dam Age	Birth Month	Birth Type	Sex	Weaning Month	Weaning Age	
2022	1	1	February	Singleton	Male	June	79–110 days	1
2021	2	2	March	Twins	Female	July	110–130 days	2
2020	-	3	April	-	-	-	130–140 days	3
2019	-	4	-	-	-	-	>140 days	4
2018	-	-	-	-	-	-	-	5

“-” indicates “none”.

**Table 3:** Fixed effects tests (F-values) for Birth Weight (BWT) in Yunnan semi-fine wool sheep.

Factor	Degrees of Freedom	Type III Sum of Squares	Mean Square	F-Value
Year	4	39.63 **	9.90	25.17
Sex	1	20.43 **	20.43	51.90
Farm	1	0.00 ^NS^	0.00	0.00
Herd Group	9	47.02 **	5.22	13.27
Litter Size	1	339.70 **	339.70	862.90
Dam Age	3	79.06 **	26.35	66.95
Lambing Month	2	9.68 **	4.84	12.31

** *p* < 0.01. NS: non-significant (*p* > 0.05).

**Table 4:** Least squares analysis of variance for Birth Weight (BWT) of Yunnan semi-fine wool sheep.

Birth Year	*n*	LSM ± SE	Dam Age	*n*	LSM ± SE
2022	819	4.05 ± 0.05 ^bc^	1	786	3.86 ± 0.05 ^b^
2021	598	4.32 ± 0.05 ^a^	2	1283	4.25 ± 0.05 ^a^
2020	557	4.03 ± 0.05 ^c^	3	903	4.26 ± 0.05 ^a^
2019	709	4.10 ± 0.05 ^bc^	4	280	4.24 ± 0.06 ^a^
2018	569	4.26 ± 0.05 ^ab^	Herd Group	*n*	LSM ± SE
Sex	*n*	LSM ± SE	1	444	4.24 ± 0.05 ^b^
Male	1645	4.07 ± 0.05	2	346	4.21 ± 0.06 ^b^
Female	1607	4.23 ± 0.05	3	293	3.9 ± 0.05 ^d^
Farm	*n*	LSM ± SE	4	314	3.98 ± 0.05 ^c^
Laishishan	2643	4.15 ± 0.04	5	246	3.92 ± 0.06 ^c^
Xiaohai	609	4.15 ± 0.05	6	388	4.23 ± 0.05 ^b^
Birth Type	*n*	LSM ± SE	7	430	4.22 ± 0.05 ^b^
Singleton	2720	4.61 ± 0.04	8	374	4.13 ± 0.05 ^b^
Twins	532	3.70 ± 0.05	9	366	4.21 ± 0.05 ^b^
Birth Month	*n*	LSM ± SE	10	51	4.47 ± 0.10 ^a^
February	2075	4.05 ± 0.02 ^b^	-	-	-
March	1156	4.19 ± 0.02 ^a^	-	-	-
April	21	4.22 ± 0.13 ^a^	-	-	-

The data in the same column with different lowercase letters indicate significance (*p* < 0.05), while identical letters suggest no significance (*p* > 0.05), “-” indicates “none”.

**Table 5:** Fixed effects tests (F-values) for Weaning Weight (WWT) in Yunnan semi-fine wool sheep.

Factor	Degrees of Freedom	Type III Sum of Squares	Mean Square	F-Value
Year	4	17,381.09 **	4345.27	236.41
Sex	1	1569.98 **	1569.98	85.42
Farm	1	4.23 ^NS^	4.23	0.23
Herd Group	9	5256.81 **	584.09	251.55
Litter Size	1	5700.75 **	5700.75	246.09
Dam Age	3	2712.35 **	904.11	49.19
Weaning Month	1	180.84 *	180.84	9.84
Weaning Age Days	3	2712.35 **	904.11	49.19

** *p* < 0.01, * *p* < 0.05. NS: non-significant (*p* > 0.05).

**Table 6:** Least squares analysis of variance for Weaning Weight (WWT) of Yunnan semi-fine wool sheep.

Birth Year	*n*	LSM ± SE	Weaning Month	*n*	LSM ± SE
2022	819	18.48 ± 0.18 ^c^	June	2869	20.60 ± 0.20
2021	598	25.89 ± 0.20 ^a^	July	383	22.33 ± 0.30
2020	557	20.50 ± 0.22 ^b^	Weaning age days	*n*	LSM ± SE
2019	709	21.16 ± 0.22 ^b^	79–110 days	640	19.33 ± 0.23 ^c^
2018	569	21.28 ± 0.25 ^b^	110–130 days	1580	20.94 ± 0.17 ^b^
Sex	*n*	LSM ± SE	130–140 days	794	22.51 ± 0.20 ^a^
Male	1645	20.77 ± 0.16 ^a^	>140 days	238	23.08 ± 0.29 ^a^
Female	1607	22.16 ± 0.16 ^b^	Herd Group	*n*	LSM ± SE
Farm	*n*	LSM ± SE	1	444	21.90 ± 0.24 ^bc^
Laishishan	2643	21.65 ± 0.24	2	346	23.43 ± 0.27 ^a^
Xiaohai	609	21.27 ± 0.21	3	293	20.44 ± 0.27 ^e^
Birth Type	*n*	LSM ± SE	4	314	20.56 ± 0.27 ^de^
Singleton	2720	23.33 ± 0.14	5	246	21.42 ± 0.30 ^cd^
Twins	532	19.60 ± 0.20	6	388	22.42 ± 0.25 ^ab^
Dam Age	*n*	LSM ± SE	7	430	19.36 ± 0.25 ^e^
1	786	19.41 ± 0.20 ^b^	8	374	22.37 ± 0.24 ^ab^
2	1283	22.27 ± 0.16 ^a^	9	366	20.38 ± 0.26 ^de^
3	903	22.23 ± 0.18 ^a^	10	51	22.33 ± 0.61 ^ab^
4	280	21.94 ± 0.27 ^a^	-	-	-

The data in the same column with different lowercase letters indicate significance (*p* < 0.05), while identical letters suggest no significance (*p* > 0.05), “-” indicates “none”.

**Table 7:** Variance components for Birth Weight (BWT) estimated by different animal models.

Model	$\sigma_{\alpha }^{2}$	$\sigma_{m}^{2}$	$\sigma_{c}^{2}$	$\sigma_{e}^{2}$	$\sigma_{p}^{2}$	$h_{\alpha }^{2}$	$h_{m}^{2}$	$h_{c}^{2}$	$\sigma_{am}$
1	0.1289	-	-	0.2620	0.3909	0.3298	-	-	-
2	0.1034	-	0.0504	0.2354	0.3892	0.2657	-	0.1295	-
3	0.1015	0.0470	-	0.2413	0.3898	0.2605	0.1205	-	-
4	0.1162	0.0643	-	0.2280	0.3722	0.3123	0.1726	-	−3.63 × 10^−2^
5	0.1249	0.1034	0.0504	0.2354	0.5142	0.2430	0.2011	0.0980	-
6	0.2322	0.1034	0.0504	0.2355	0.6065	0.3828	0.1704	0.0831	−1.49 × 10^−2^

$\sigma_{\alpha }^{2}$: direct genetic variance; $\sigma_{m}^{2}$: mother genetic variance; $\sigma_{e}^{2}$: residual variance; $\sigma_{c}^{2}$: mother permanence environmental variance; $\sigma_{am}$: direct and mother genetic covariance; $\sigma_{p}^{2}$: phenotypic variance; $h_{\alpha }^{2}$: direct genetic effect; $h_{m}^{2}$: mother genetic effect; $h_{c}^{2}$: mother permanence environmental effect; “-”: without this effect in the model.

**Table 8:** Estimation of variance components for Weaning Weight (WWT) using different animal models.

Model	$\sigma_{\alpha }^{2}$	$\sigma_{m}^{2}$	$\sigma_{c}^{2}$	$\sigma_{e}^{2}$	$\sigma_{p}^{2}$	$h_{\alpha }^{2}$	$h_{m}^{2}$	$h_{c}^{2}$	$\sigma_{am}$
1	5.8876	-	-	11.9501	17.8377	0.3301	-	-	-
2	6.0876	-	2.2762	9.1744	17.5382	0.3471	-	0.1298	-
3	5.5776	1.6255	-	9.5990	16.8021	0.3320	0.0967	-	-
4	6.0876	2.2762	-	3.5083	9.1744	0.6635	0.2481	-	1.52 × 10^−2^
5	2.7988	5.6967	1.7224	9.3686	19.5865	0.1429	0.2908	0.0879	-
6	2.8152	5.6934	1.7236	9.3704	19.6062	0.1436	0.2904	0.0879	3.57 × 10^−3^

$\sigma_{\alpha }^{2}$: direct genetic variance; $\sigma_{m}^{2}$: mother genetic variance; $\sigma_{e}^{2}$: residual variance; $\sigma_{c}^{2}$: mother permanence environmental variance; $\sigma_{am}$: direct and mother genetic covariance; $\sigma_{p}^{2}$: phenotypic variance; $h_{\alpha }^{2}$: direct genetic effect; $h_{m}^{2}$: mother genetic effect; $h_{c}^{2}$: mother permanence environmental effect; “-”: without this effect in the model.

**Table 9:** Standard values of AIC information for traits in different animal models.

Model	BWT		WWT	
	−2logL	AIC	−2logL	AIC
1	201.50	205.50	12,543.80	12,547.80
2	158.27	164.27	12,291.67	12,297.67
3	162.66	168.66	12,298.77	12,304.77
4	151.98	159.98	12,291.67	12,299.67
5	153.82	161.82	12,303.05	12,311.05
6	155.23	165.23	12,303.04	12,313.04

**Table 10:** Likelihood ratios and χ2 test results for different model comparisons of each trait.

Model Comparison	df	BWT	WWT
1 vs. 2	1	43.2314 **	252.1310 **
1 vs. 3	1	38.8369 **	245.0297 **
1 vs. 4	2	49.5147 **	252.1310 **
1 vs. 5	2	47.6751 **	240.7535 **
1 vs. 6	3	46.2666 **	240.7673 **
2 vs. 5	1	4.4437 *	11.3776 **
2 vs. 6	2	3.0352 ^NS^	11.3638 **
3 vs. 4	1	10.6779 **	7.1014 **
3 vs. 5	1	8.8383 **	4.2762 *
3 vs. 6	2	7.4298 *	4.2624 *
4 vs. 6	1	3.2481 *	11.3638 **
5 vs. 6	1	1.4085 ^NS^	0.0138 ^NS^

** *p* < 0.01, * *p* < 0.05; NS means no significance.

## Data Availability

The data presented in this study are available from the corresponding authors upon reasonable request. The data are not publicly available due to privacy or ethical restrictions.

## Abbreviations

The following abbreviations are used in this manuscript:

BWT	Birth Weight
WWT	Weaning Weight
